# Supplementary material for: Immunization with peptide encapsulated within synthetic spores activates T cell responses and reduces tumor growth
Source: bioRxiv. 2025 Feb 27:2025.02.27.640614. Preprint. [Version 1] doi: 10.1101/2025.02.27.640614 (PMC12190748; doi:10.1101/2025.02.27.640614)
Supplement: 1 [file NIHPP2025.02.27.640614v1-supplement-1.pdf]

bioRxiv preprint doi: https://doi.org/10.1101/2025.02.27.640614; this version posted February 27, 2025. The copyright holder for this preprint (which was not certified by peer review) is the author/funder. This article is a US Government work. It is not subject to copyright under 17 USC 105 and is also made available for use under a CC0 license.

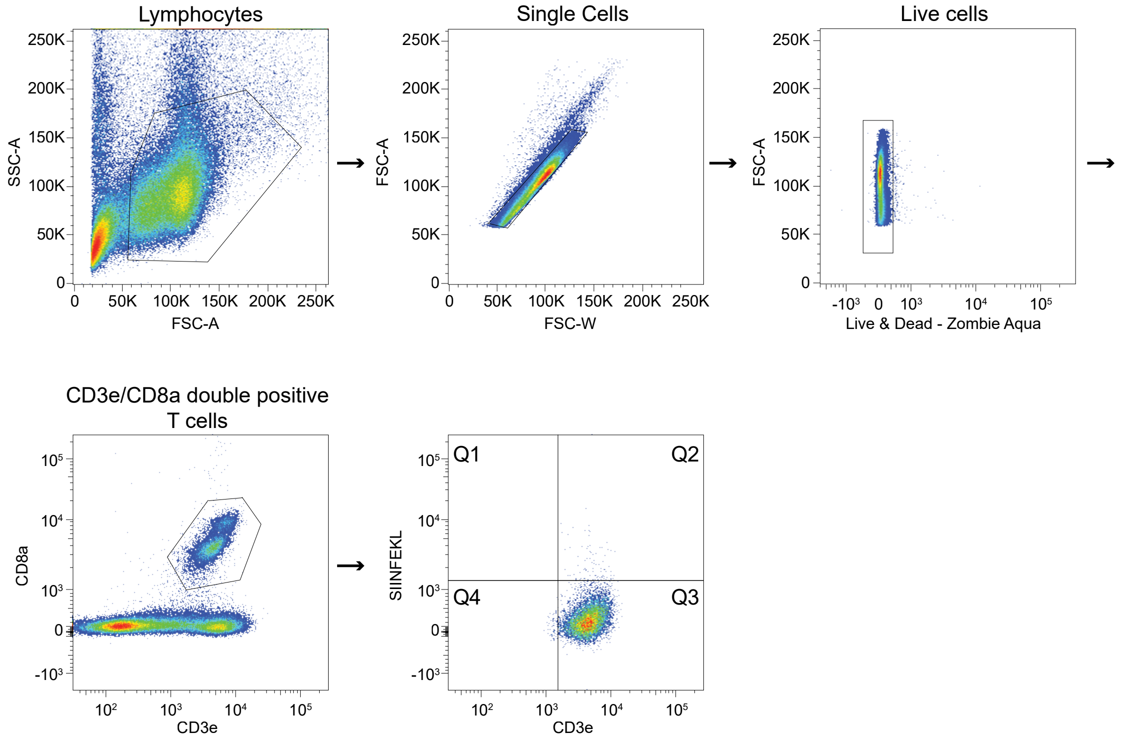

Figure S1. Gating strategy to isolate CD3+/CD8+ cytotoxic T cells that recognize the SIINFEKL epitope, using the H-2Kb tetramer. Related to Fig. 4.

bioRxiv preprint doi: https://doi.org/10.1101/2025.02.27.640614; this version posted February 27, 2025. The copyright holder for this preprint (which was not certified by peer review) is the author/funder. This article is a US Government work. It is not subject to copyright under 17 USC 105 and is also made available for use under a CC0 license.

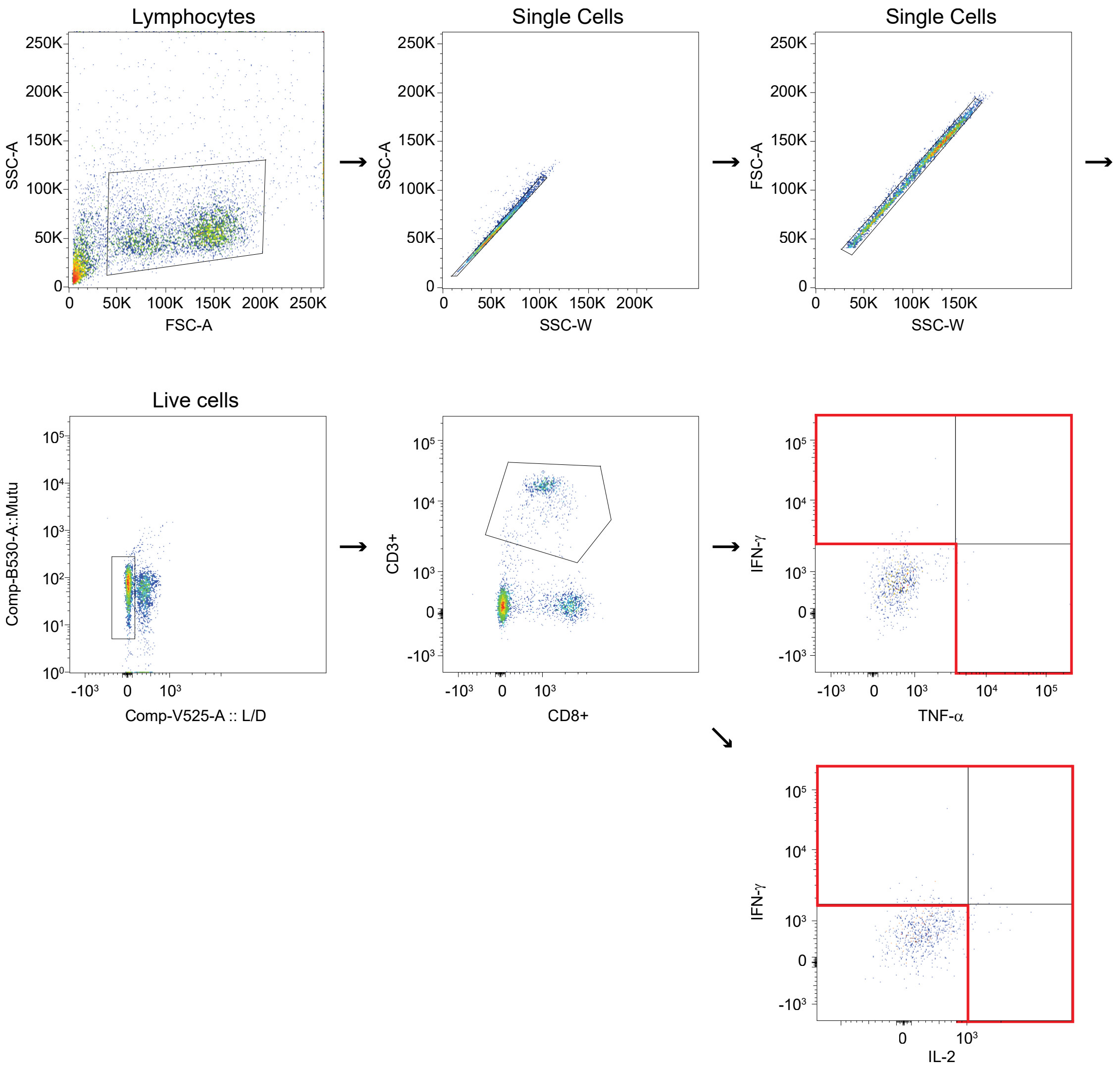

Figure S2. Gating strategy to isolate cytotoxic T cells releasing the indicated cytokines.

Related to Fig. 5.

bioRxiv preprint doi: https://doi.org/10.1101/2025.02.27.640614; this version posted February 27, 2025. The copyright holder for this preprint (which was not certified by peer review) is the author/funder. This article is a US Government work. It is not subject to copyright under 17 USC 105 and is also made available for use under a CC0 license.

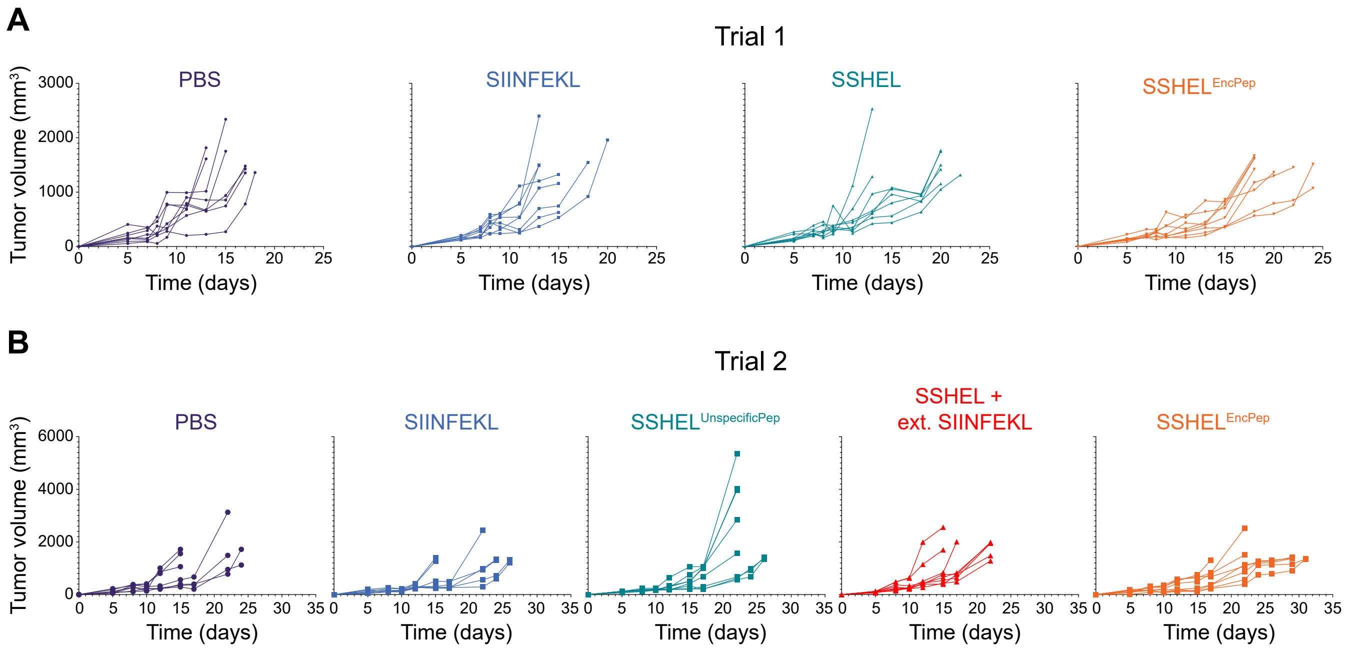

Figure S3. Mean weight of mice in immunization trials. (A) Trial 1 and (B) Trial 2. Data points represent mean; errors are S.D. Related to Fig. 6.

bioRxiv preprint doi: https://doi.org/10.1101/2025.02.27.640614; this version posted February 27, 2025. The copyright holder for this preprint (which was not certified by peer review) is the author/funder. This article is a US Government work. It is not subject to copyright under 17 USC 105 and is also made available for use under a CC0 license.

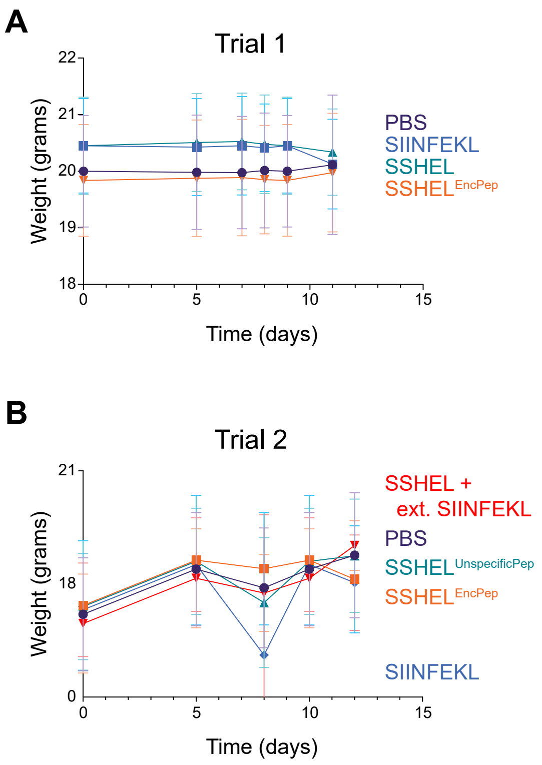

Figure S4. Individual tumor sizes of mice in immunization trials. (A) Trial 1 and (B) Trial 2. Treatment groups are indicated above the graphs.

bioRxiv preprint doi: https://doi.org/10.1101/2025.02.27.640614; this version posted February 27, 2025. The copyright holder for this preprint (which was not certified by peer review) is the author/funder. This article is a US Government work. It is not subject to copyright under 17 USC 105 and is also made available for use under a CC0 license.

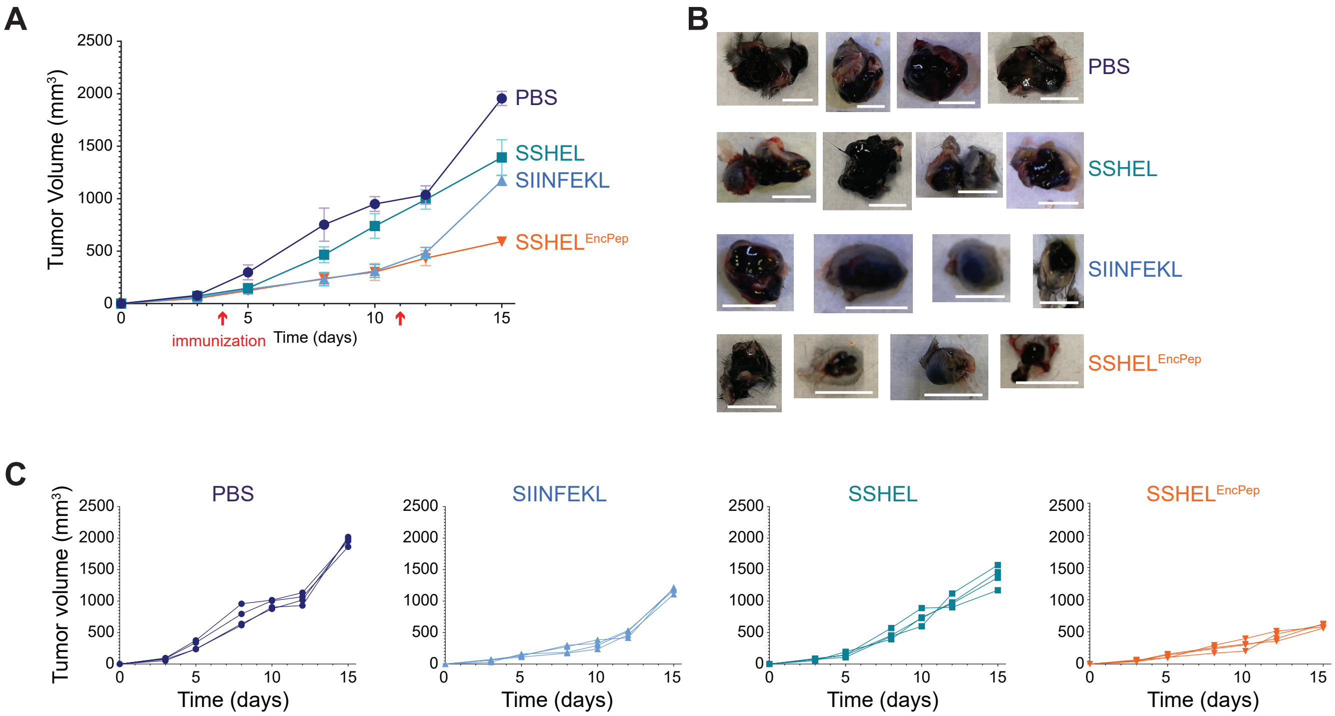

Figure S5. Trial 3 to assess tumor pathology. (A) Subcutaneous B16-N4-OVA tumor was introduced in C57Bl/6 mice (4 mice/treatment group). When tumors reached ~100 mm^3, the first inoculation was administered (7 µg peptide), followed by a second inoculation two weeks later, and tumor size was periodically measured. Mice were treated with PBS (purple), SIINFEKL peptide alone (blue), SSHEL alone (green), or SSHEL^{EncPep} (orange). (B) Images of resected tumors. Size bars: 10 mm. (C) Individual tumor sizes of each mouse.
